# Piloting a new approach in primary care to identify, assess and support carers of people with terminal illnesses: a feasibility study

**DOI:** 10.1186/s12875-016-0414-2

**Published:** 2016-02-11

**Authors:** Emma Carduff, Alison Jarvis, Gill Highet, Anne Finucane, Marilyn Kendall, Nadine Harrison, Jane Greenacre, Scott A Murray

**Affiliations:** Marie Curie Hospice, Balornock Rd, Glasgow, G33 3US UK; NHS Lothian, Waverley Gate, 2-4 Waterloo Place, Edinburgh, EH1 3EG UK; NHS Lothian, New Royal Infirmary Edinburgh, 51 Little France Crescent, Edinburgh, EH16 4SA UK; Marie Curie Hospice Edinburgh, Frogston Road West, Edinburgh, EH10 7DR UK; Primary Palliative Care Research Group, Centre for Population Health Sciences, The Usher Institute, The University of Edinburgh, Medical School, Teviot Place, Edinburgh, EH8 9AG UK; Voice of Carers Across Lothian, 8-13 Johnston Terrace, Edinburgh, Midlothian EH1 2PW UK

**Keywords:** End of life, Family carer, Identification, Informal carer, Lay carer, Palliative care, Primary care, Support

## Abstract

**Background:**

General practices in the United Kingdom are encouraged to have a protocol for the identification of carers and a mechanism for social care referral. However, a minority of carers are identified and those caring for someone with a terminal illness often cope until the situation becomes overwhelming. Earlier identification could enable more timely support.

The aim of this project was to model and pilot a systematic approach to identify, assess and support carers of people with supportive and palliative care needs in primary care.

**Method:**

The intervention was modelled on the Medical Research Council complex intervention framework with a preliminary theoretical phase, which has been reported elsewhere. In this study, which lasted 12 months, four general practices were recruited. Each practice identified a ‘carer liaison’ person to take the lead in identifying carers, followed by assessment and support using a toolkit modelled from the earlier phase. Qualitative evaluation interviews were conducted with carers who had received the intervention and the carer liaisons and general practitioners in the pilot practices. A stakeholder event was held to disseminate and deliberate the findings.

**Results:**

The practices’ populations ranged from 5840 to 10832 patients and across the four practices, 83 carers were identified. Thirty six carers were identified from practice registers (disease - 16; palliative care - 9; carer - 11; advanced care plan - 12), whilst 28 were identified opportunistically by practice staff at appointments or at home. Seven carers self-identified. Overall, 81 carers received the carer pack and 25 returned the Carer Support Needs Assessment Tool (CSNAT) form. Eleven carers received a follow up call from the practice to discuss support and 12 were also referred/signposted for support. Qualitative interviews suggest carers valued connection with their practices but the paperwork in the toolkit was onerous.

**Conclusion:**

This approach to identifying and supporting carers was acceptable, but success was dependent on engagement within the whole practice. Carers did not tend to self-identify, nor ask for help. Practices need to proactively identify carers using existing opportunities, resources and computer systems, and also adopt a public health approach to raise carer awareness and perceived support within their communities.

## Background

Approximately 17 % of the Scottish population provide unpaid care for a relative, friend or neighbour [1]. Although caring can be a positive and rewarding experience, it can also have detrimental effects on the health and wellbeing of the carer. This can subsequently impact on the well-being of the person they care for. Caring can exacerbate pre-existing health problems or lead to new health problems for the carer [1]. 32 % of carers have indicated that caring has had a negative impact on their health with the biggest impact being psychological [1]. In a recent survey, unpaid carers reported poorer quality of life than non-carers [2]. The Scottish Health Survey analysis of mental well-being amongst carers showed that mental wellbeing scores decreased as hours of care provided increased [1, 3]. Carers who are supporting someone at the end of life are more likely to suffer depression [4], social isolation, stress, anxiety and all of this in the context of losing someone close to them [5]. Around 70 % of carers receive no support with caring and, even among the 42 % of people who provide more than 35 hours of care per week [1]. Those who provide high levels of care over extended periods, are most at risk of poor mental health [1].

Previous work undertaken at the start of this study showed that one of the reasons carers did not identify themselves was that they were engulfed in their caring responsibilities and were unable to consider their own needs [6]. Carers UK (2014) found that carers were less likely to actively engage in health promoting behaviours such as exercise and maintaining a balanced diet because of their caring responsibilities [7].

The primary health care team (PHCT), is well placed to identify carers [8]. General practices are encouraged to have a protocol for identifying carers, but most carers still go unrecognised. The benefit to the individual carer of being on the carer register varies: some practices use the register to offer carers annual flu vaccinations, longer and more flexible appointments as well as additional health checks. One of the major challenges of identifying carers in primary care is that neither carers, nor professionals see carers' needs as paramount. Caring, per se, is not seen as a medical problem [6].

Addressing carers’ needs for respite, information, advice and training are effective ways to support carers – but if carers are not identified they are unlikely to access even small amounts of support that might make a difference. However, even when carers are known to professionals, they are often unaware of available support. [6, 9] Caring for someone with a terminal illness is particularly challenging [5, 10–12], therefore it is all the more important that such carers are identified early and signposted to support – recognising the importance of a preventative approach for carers at an early stage of caring when the demands may be less intensive.

There is now an increasing number of unpaid carers, in the light of an ageing population with more complex needs. Many of these carers will be supporting someone with a progressive illness. This study was designed to develop, pilot and evaluate a new model of identifying, assessing and supporting unpaid carers of people with palliative and supportive care needs, which could be further trialled in UK primary care.

## Methods

This feasibility study followed the Medical Research Council (MRC) guidelines for developing complex interventions and was conducted over 2 phases [13, 14]. We gained ethical approval for the study from South East Scotland Research Ethics Committee (REC reference 12/SS/0142). A lay advisory group of carers was recruited in the first month of the study and met quarterly throughout to advise the multidisciplinary research team.

### Phase 1 - Developing the model

In phase 1 we triangulated the findings from a literature review, focus groups and our previous extensive research involving carers of people at the end-of-life. We set out to understand the barriers to identifying carers from the perspective of carers themselves, the people they care for and health professionals. The findings from phase 1 have been reported elsewhere [6].

### Phase 2 - Defining the model in practices

#### Recruitment of the practices

Four practices were selected and recruited to reflect heterogeneity in practice size and demographics. These practices were known to the carer organisation who collaborated on the study (JG) and were thought to be ‘carer aware’, but that there was room for improvement in terms of identification and support. We approached the practice manager and General Practitioners (GPs) to discuss how the study would work and consulted them about the intervention before finalising the model. The intervention ran for 12 months in each practice.

#### Conducting the intervention

**Fig. 1:**
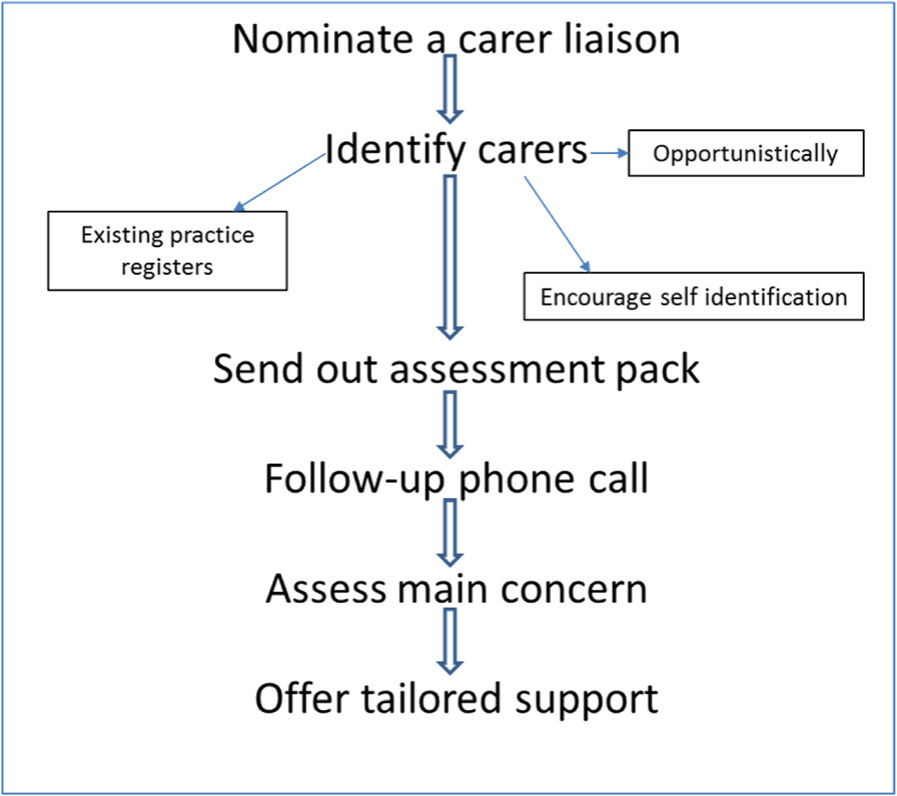
Process of the new approach to identifying, assessing and supporting carers. This process of identifying, assessing and supporting carers was derived as a result of the findings from phase 1 of the project [6] and in consultation with the practices

Based on the findings from phase 1, and in consultation with the practices and our lay advisory group, we developed a new practical approach to identification, assessment and support. Figure 1 shows the process of the new model. First we suggested practices nominate a named carer liaison person (who they thought most appropriate) to lead the project. The research team met with the carer liaison regularly as the model was being rolled out and they were given detailed information on the intervention. We then suggested practices search existing registers (carer, disease specific registers such as dementia and other long term conditions, palliative care, advanced care plan (ACP)) to identify carers. Practices also displayed a poster in the waiting room to encourage carers to self-identify. When a carer was identified they were sent a carer toolkit which included:

- A cover letter on practice headed notepaper, explaining that the practice would like to support any unmet needs they might have.
- A brief questionnaire requesting demographic details about the carer and details of the extent of their caring role.
- The Carer Support Needs Assessment Tool (CSNAT) [15].
- ‘Who to call?’ fridge magnet with useful numbers for those approaching the end of life.

When the carer liaison sent out a carer toolkit they completed an audit form which was anonymised, photocopied and sent to the research team. If the assessment pack was returned and support needs were identified on the CSNAT, carers were invited to a follow-up conversation with the carer liaison (telephone or face to face). Carers were given the option not to be contacted. During the follow-up conversation, carers were offered/signposted to relevant support. A carer toolkit was also available for carers who were not registered in the pilot practices where the person cared for was registered. This included a cover letter, questionnaire and a leaflet for a local carer organisation.

#### Evaluating the model

We conducted semi-structured qualitative interviews with 11 carers who had received the intervention from their practice and with the carer liaison and one GP in each practice (total = 19). The carer interviews were conducted in the carer’s own home or by telephone. Written consent was sought and the interviews were audio-recorded, transcribed and entered into the qualitative data analysis software package, NVivo [16]. All four carer liaison interviews were conducted in the GP practices. Telephone interviews were conducted with GPs. The data were then coded thematically and analysed using a coding framework devised by both researchers on the project (EC and GH). Emerging findings were discussed at regular intervals with the research team to ensure rigour. Specifically, the analysis focused on exploring the impact of the caring role on carers’ lives, potential barriers to/strategies for identifying and supporting carers, carers’ views on the role of the intervention in meeting their support needs, challenges associated with supporting carers, and future potential for providing carers’ support in a primary care context.

We also convened a stakeholder event at the end of the project to discuss, formulate and disseminate our key findings with 21 health care professionals, academics and carers. The event was designed to disseminate, discuss and deliberate our findings. The group made recommendations based on the main findings of the study and some of these feature in the discussion.

## Results

Table 1 shows demographic information for each practice, pre and post intervention. All 4 practices had a protocol to identify carers. The primary care team member assigned to be the carer liaison in each practice differed. Three had a clinical role and one an administration role.

**Table 1:** Demographic information for each practice - Pre and post intervention

	Practice A		Practice B		Practice C		Practice D	
	Pre	Post	Pre	Post	Pre	Post	Pre	Post
No of patients	10832	10464	5840	5601	9122	9156	7044	7112
Role of carer liaison	Health care assistant		Practice manager/nurse		Administrator/data entry		Phlebotomist	
No of carers on carer register (all carers)	224 (2%)	241 (2.3%)	41 (0.7%)	41 (0.7%)	19 (0.2%)	17 (0.2%)	92(1.3%)	112 (1.6%)
How do carers get on the register?	Questionnaire/GP or nurse identify/flu clinics	Questionnaire; GP/nurse identify/flu clinics	Referral/posters in waiting room/new patients/ad hoc	Referral/posters in waiting room/new patients/ad hoc	Referral from GPs or DNs	New patient registration form/GP’s/DNs	Clinician suggestion/staff ID/in-house invite e.g. registration forms	Registration/GPs, nurses at consultation/Flu season

### Intervention data

**Fig. 2:**
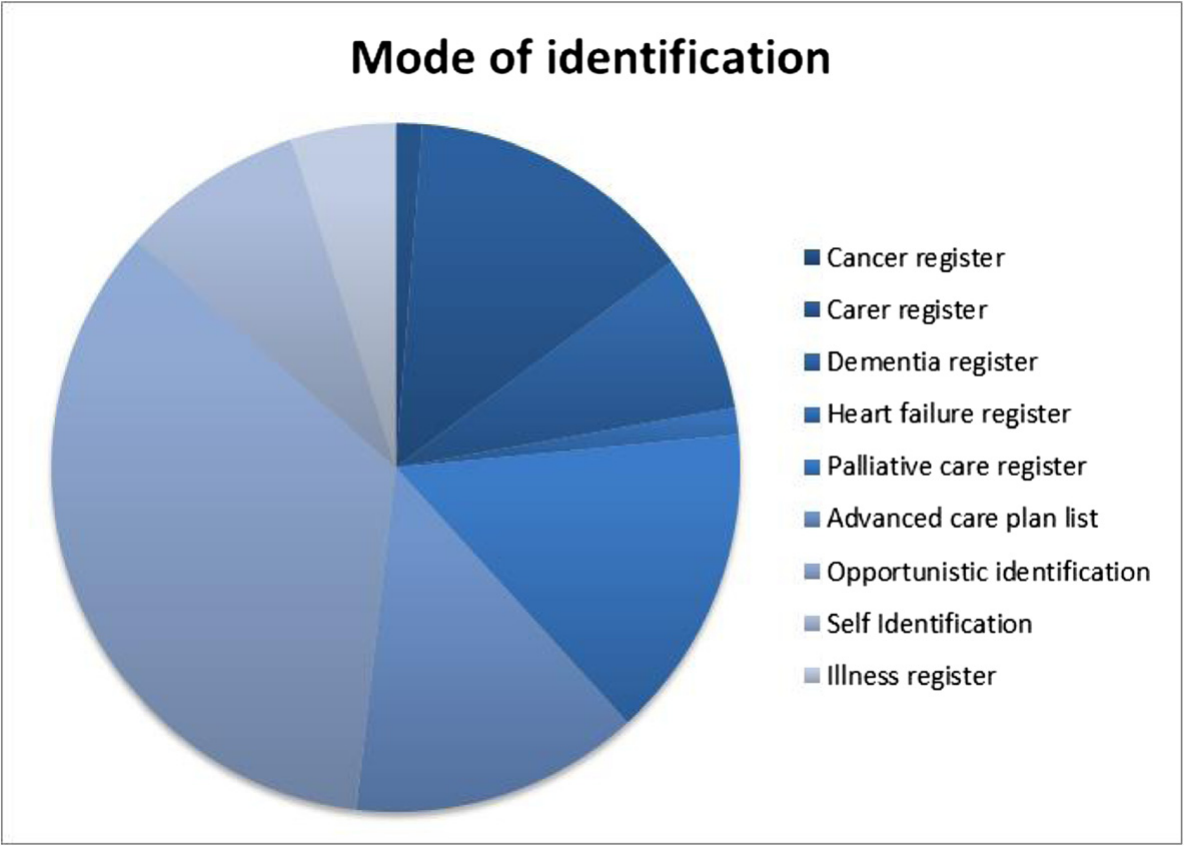
Mode of identification. The various modes of identifying carers were developed in consultation with the practices. Figure 2 illustrates how many carers were identified using each mode of identification. Audit forms which were kept by the carer liaison in each practice then forwarded to the researcher showed that overall, 83 carers were identified using 6 different mechanisms (Anticipatory care plans (*n* = 12), carer register (*n* = 11), illness registers (*n* = 16), opportunistically (*n* = 28), palliative care register (*n* = 9) and via self-identification in the practice (*n* = 7))

Identification - Fig. 2 shows how carers were identified. Carer liaisons identified carers through practice registers, opportunistically during a routine appointment or carers could self-identify in the practice. In total, 83 patients were identified as having a caring role. Twenty eight of the 83 carers were identified opportunistically by the carer liaison or a GP. Thirty-six carers were identified from practice registers: palliative care register (*n* = 9), illness registers (*n* = 16), carer register (*n* = 11). Twelve carers were identified through patient Anticipatory Care Plans (ACP) of the cared for person which we encouraged practices to also consider (ACPs were the subject of a new directed enhanced service (DES) in Scatland at the time of the study). Posters were put up in the practices at the beginning of the intervention to encourage carers to self-identify but only seven carers did so. Twelve carers were registered in another practice and as a result were not given the CSNAT to complete.

**Fig. 3:**
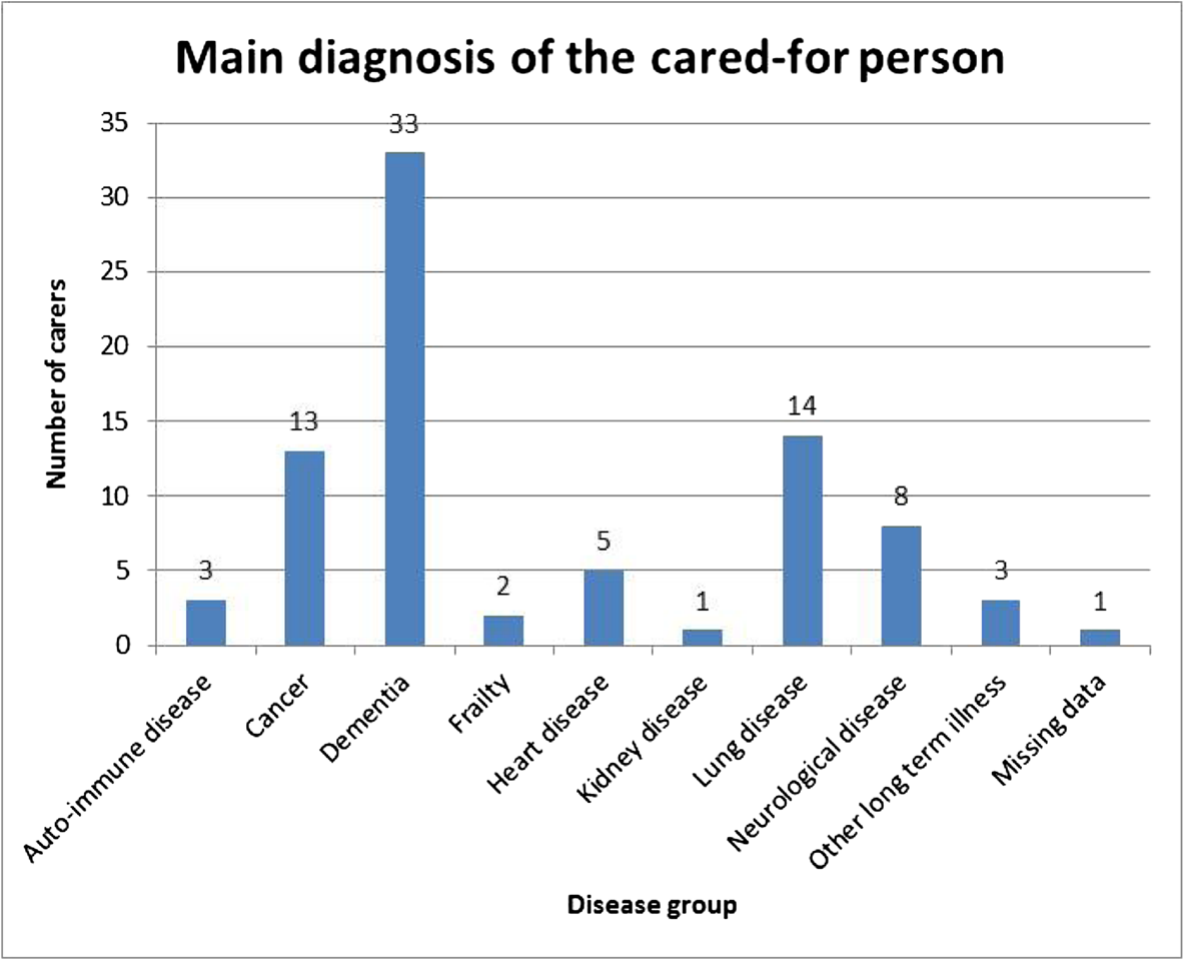
Main diagnosis of the cared-for person. The bar chart in Fig. 3 shows the diagnosis of the cared for person for the 83 carers who were identified. Data were collected from the audit forms which were kept by the carer liaison in each practice. Most of the carers identified by the carer liaison were caring for someone with dementia (*n* = 33). Other diagnoses included auto-immune disease (*n* = 3), cancer (*n* = 13), frail older people (*n* = 2), heart disease (*n* = 5), kidney disease (*n* = 1), lung disease (*n* = 14) and neurological disease (*n* = 8). The category of other diagnoses included chronic alcoholism and late stage Downs Syndrome (*n* = 3)

Of the 83 carers identified, 55 (66 %) were female and 28 (34 %) male. Figure 3 shows the main diagnoses of the cared for people. Thirty three carers were caring for a person with dementia (40 %), while 13 (16 %) carers were caring for a person with cancer and the same for lung disease.

Assessment and support - Table 2 shows the return data for the carer assessment packs. Thirty packs were completed and returned. Twenty-five returned the CSNAT and of these, 20 identified at least 1 need. Follow-up conversations between the carer liaison and carer were conducted with 18 of the 20 who reported need. Of these, 12 were signposted or referred for support. Eleven were directed to Voice of Carers across Lothian (VOCAL) and others included the local Maggie’s Centre, the GP, Alzheimer’s Scotland, Volunteer Net, and Edinburgh Carer Council. Seven carers were referred to more than one place.

**Table 2:** Return data for the carer assessment packs

Outcome	Number
Assessment packs sent	81
Packs completed and returned	30
CSNAT returned	25
Carers identified at least 1 need	20
Needs were identified on average	4 (1-11)
Follow-up conversations	18
Carers signposted/referred for support	12

### The acceptability of the intervention

#### Carers

Eleven carers, with a mean age of 74 years (range 58 years – 86 years), were interviewed across the participating practices. Table 3 shows the age, relationship and illness of the cared for person for the caregivers. Eight were caring for a close relative (usually a spouse) with dementia. Other conditions included cancer and lung disease. Our interviews revealed that taking on the care of someone with an advanced illness is often a gradual process, consequently many people caring for a close relative do not identify themselves as ‘carers’, preferring instead to view their caring role as a natural extension of their relationship with the cared for person.

**Table 3:** Characteristics of carer interviewees

Age	Sex of carer	Relationship to cared for person	Cared for person’s illness
58	M	Husband	Neurological
80	M	Husband	Dementia
72	F	Daughter	Dementia
86	F	Sister	Dementia
72	F	Wife	Dementia
78	F	Wife	Lung disease
77	F	Wife	Dementia
79	M	Husband	Dementia
68	F	Wife	Cancer
77	M	Husband	COPD
63	F	Sister	Cancer

“The challenge was sometimes getting people to talk to you, or to accept their role as a carer” (Carer Liaison)

This was also identified by the health professionals who described this as a barrier to engaging carers in the project.

“I guess a lot of people looking after people don’t really see themselves as a carer” (GP)

Carers of people with dementia, in particular, spoke in bleak terms about the unrelenting impact on their lives of caring for a loved one,

*“*our whole lives have just collapsed, we’ve crashed and burned*”* (Carer)

For some, feelings of loss can also extend to friends and other family members who may become distant.

“I’ve also found that nobody wants to know your problems – you lose friends, they don’t want to come to the house, they don’t want to see you, and all the rest of it. Your family itself, it gets very distant” (Carer)

Attempting to assess carers’ needs using a written assessment tool was not particularly effective in our feasibility study, partly because such forms may ‘get lost’ in the volume of paperwork that carers receive.

“I’ve got a heap of papers that I need to get through, so I probably haven’t done anything with it” (Carer)“It’s very difficult for folk to get through all the paper-work and assimilate and bring it down to what their needs are” (Carer)

Carers did not describe the need for intensive support, preferring smaller interventions. However, feeling ‘connected’ was very important to the well-being of the carers in our sample and they endorsed the provision of such support being available in their community through their local GP practice.

“It’s always nice if somebody rings you up and says, how are you? I think that would make you feel that you were connected because at the moment I don’t” (Carer)

#### Professionals

 Interviews with the Carer Liaisons and GPs in the participating sites highlighted the importance of carer support being embedded across whole practices. In GP practices in Scotland, electronic Key Information Summaries (eKIS) (a shared national electronic record to enable sharing of clinical information between unscheduled care staff and GPs [17]) may be a useful way of identifying carers as part of the anticipatory care planning process, but this may have to be championed by a key worker in the practice.

“Key Information Summary, so it’s sharing information with the out of hours, but if you start one of those they’re asking for relatives and next of kin and carer information, so it’s highlighting that sort of information that you need to be providing as well so I guess we’re picking up a lot more than we used to and we’re certainly thinking about it a lot more” (GP)

GPs were positive about the need to identify and support carers. They felt that it was part of their job as family doctors and that their relationship with families enabled carer support. Professionals also highlighted that the structure of general practice - offering continuity and holistic care - enabled carer support. GPs described that all members of staff could know patient and carer circumstances, and may flag up difficulties, but that did not necessarily require formal assessment.

“one of the advantages of continuity of care is that you get to know people and you get to know what’s going on in the wider family or at what point you need to step in and say actually, I think you need a bit of a rest and so on” (GP)

Professionals described the challenge of addressing the carer’s need in a consultation with the cared-for person. However, they also acknowledged that such a consultation could also present an opportunity to identify someone who has a caring role and is struggling.

“people might come in specifically to ask if there’s any help, or quite often come in just kind of stressed and depressed or anxious or whatever and when you go in to why that’s happening, it turns out they’ve got somebody that they’re worried about or having to spend a lot of time and effort trying to deal with and finding it difficult” (GP)

They offered some conflicting views about the public awareness of the need to support carers. Some stated that this was a current topic of public debate while others felt it should be made so. Overall, there was agreement for the need to increase awareness of what support is available to carers through signposting and public health campaigns.

“I think it’s all about information and just making sure that information is local and up to date” (GP)

However, there was frustration that GPs could identify the support which was required but that it was slow to initiate.

“it gets critical when, you know, you really need to get some kind of respite care and that side of it is really, really slow to sort of move…….if you can see that someone could really do with going into a care home or something just to give their partner a rest that actually is really hard to organise, or doesn’t happen quickly” (GP)

Overall, practices were open to new or adapted ways of working to help support carers, pleased to have been given the opportunity to consider carer support and keen to hear about the outcome of the study.

“It’s a very, very complex area but I’m so glad you’ve done this because I think it’s really made us think about it and we have discussed at partners’ meetings and its thrown up some interesting thoughts on it” (Carer Liaison)

## Discussion

We set out to develop an intervention model of carer identification, assessment, support and referral and then to pilot and evaluate the resulting intervention, focussing on identification. The findings suggest that such an intervention is both feasible and acceptable but the number of carers identified was small. In two of the practices the number of carers on the register increased, although this is an unreliable marker as carers were also removed from the register if they no longer had a caring role. Our study reflects the problematic nature of carer identification [6], in that many carers do not think of themselves as such, meaning they are unlikely to come forward or seek help. Additionally, caring for someone towards the end of life is all encompassing, meaning carers struggle to find the time to assess their own needs or ask for help [6]. In practice terms, we propose that carers should not only take part in Advance Care Planning discussions, but their own needs should be considered as well as the patient’s, using initiatives such as eKIS [17] and Co-ordinate my Care [18] to trigger carer identification and make this routine in primary care. Other triggers to identifying carers might include applications for power of attorney for carers of patients with dementia, or entry on the palliative care and disease registers. Although it has been suggested that carers only seek information which is situation and context specific [19], we invite primary care practitioners to consider the appropriateness of identifying a carer and potential needs around the time of diagnosis. In this way, crises in the caring situation may be reduced, or even eliminated. Recent incentives to increase advance care planning for patients with long term conditions may be a useful vehicle to help facilitate this internationally.

As the findings illustrate, opportunistic identification within the practices was successful – particularly where there was greater overall carer awareness. Therefore, we advocate for a programme of education for all staff about what services are available. Linking with the third sector may enable practices to achieve this without too much effort or money. A priority for carers is to feel ‘connected’ with their general practice - having a single point of contact, such as a named carer liaison or GP, is one way of achieving this. The findings suggest that identifying carers is more successful when the carer liaison has a clinical role, and has good interpersonal skills to form relationships with the carers. However, this is most likely to be effective when carers’ support is embraced, as an important priority, across the whole practice. It may be that the carer liaison role could usefully be developed to encompass an outreach element which would involve mapping out and linking in with community groups and networks in their area to try and identify carers and to offer information and advice. Given existing pressures on GP practices, this may be difficult to achieve unless it is incentivised financially. However, in doing so, carer support can be tailored to the needs of individual communities. For example, young carers and black, ethnic minority, lesbian, gay and transgender carers have been very hard to identify [19].

We propose that the Primary Health Care Team, possibly in conjunction with local carer organisations, actively reach out to their communities and embed carer support within a locality and as a result connect carers to both their practices and their communities. They should adopt a neighbourhood approach to raise awareness of the caring role, not just to those caring at the end of life, but to the estimated 17 % of the Scottish population who are caring. This would foster relationships between third sector organisations and encourage the transfer of knowledge. Outreach/community strategies would also encourage carers within the community to come together. Many people caring for a close relative prefer to draw on informal support from family and community rather than accessing more formal support. A recent study reported that caregivers of cancer patients found family support facilitated the search for meaning after diagnosis [20]. A number of carers in our study spoke of the importance of peer support as a way of gaining information. We should help carers to nurture the informal relationships where they find support. Community initiatives to help identify and support carers are starting to bring a health promoting approach to end of life care, and these developments can be facilitated by primary care teams using a toolkit [21, 22].

However, professionals should not assume that all carers need support. Support comes in many guises and practices should not fear asking people what help they need. The findings in this study suggest that carers do not have a great number of needs but they did value knowing whom to contact when their situation changed. The integration of health and social care in Scotland will bring about changes to carer services as they are devolved to localities, which makes this paper timely.

Our findings point to the need for an individualised, innovative and flexible approach to carer support and assessment that takes account of both social and medical needs. There is an increasing number of innovative methods of support which are shown to be acceptable and useful to carers who are supporting someone at home [23, 24]. Yet, this support can only be given if the carer is identified in the first place. Such an approach should also acknowledge different and dynamic dimensions of need across the whole carer trajectory and in relation to the illnesses of those they are caring for. For example, caring for someone with dementia is likely to be a different experience to caring for someone with cancer or COPD. In light of our discussions at our stakeholder event, we suggest that future research should explore innovative modes of identifying carers of people with terminal illness, earlier in the illness trajectory – possibly using computerised searches in primary care. Support strategies could include empowering carers to identify personal resources to facilitate coping through semi-guided conversations with the carer liaison or other carer organisation, and approaches to embedding carers identification and support across whole practices and local communities.

## Limitations

The study was only conducted in four general practices and therefore the findings are not necessarily generalizable. Although we recruited to reflect differences in size and demography, all were located in a geographically homogenous area. This project was designed to identify carers with a supporting role for someone towards the end of life. However we have become aware of the challenge of identifying when the cared-for people are at the end of life. Often, the palliative care register is used for patients who are in the last few weeks of life and for many; by that time, the opportunity to support the carers has largely gone. The first theoretical phase of this project highlighted the challenges in identifying carers. Although our intervention was designed to address the barriers, many carers remained unidentified for the complex reasons we described.

This study is strengthened by the continued presence of the lay advisory group of carers and the stakeholder event. The researcher met with the advisory group quarterly and their discussions informed the design of the project, the intervention and future work.

## Conclusion

The identification and support of caregivers is essential to ensure that an older and frailer population can be supported to live, and then die in the community. Identifying carers early may help to avoid a crisis in the caregiving situation. Practices need to proactively identify carers using existing opportunities, resources and IT systems. Early identification would also encourage an individualised and tailored program of support. Carers also find support in their informal networks and communities. A public health approach is required to enable communities to harness informal networks to improve the overall health and wellbeing of those with an unpaid caring role.
