# Erratum to: Piloting a new approach in primary care to identify, assess and support carers of people with terminal illnesses: a feasibility study

**DOI:** 10.1186/s12875-016-0439-6

**Published:** 2016-04-01

**Authors:** Emma Carduff, Alison Jarvis, Gill Highet, Anna Lloyd, Anne Finucane, Marilyn Kendall, Nadine Harrison, Jane Greenacre, Scott A. Murray

**Affiliations:** Marie Curie Hospice, Balornock Rd, Glasgow, G33 3US UK; NHS Lothian, Waverley Gate, 2-4 Waterloo Place, Edinburgh, EH1 3EG UK; NHS Lothian, New Royal Infirmary Edinburgh, 51 Little France Crescent, Edinburgh, EH16 4SA UK; Marie Curie Hospice Edinburgh, Frogston Road West, Edinburgh, EH10 7DR UK; Primary Palliative Care Research Group, Centre for Population Health Sciences, The Usher Institute, The University of Edinburgh, Medical School, Teviot Place, Edinburgh, EH8 9AG UK; Voice of Carers Across Lothian, 8-13 Johnston Terrace, Edinburgh, Midlothian EH1 2PW UK

## Erratum

Unfortunately, the original version of this article [1] contained an error. The name of one of the authors, Anna Lloyd, was not included in the author list.

The correct author list and the new “Authors’ contributions” section have been correctly included in this erratum.
